# Deletion of POMT2 in Zebrafish Causes Degeneration of Photoreceptors

**DOI:** 10.3390/ijms232314809

**Published:** 2022-11-26

**Authors:** Yu Liu, Jaclyn M. Rittershaus, Miao Yu, Rachel Sager, Huaiyu Hu

**Affiliations:** Center for Vision Research, Departments of Neuroscience and Physiology and of Ophthalmology and Visual Sciences, Upstate Medical University, Syracuse, NY 13210, USA

**Keywords:** retina, retinitis pigmentosa, photoreceptor degeneration, inherited retinal degeneration, zebrafish, congenital muscular dystrophy

## Abstract

Mutations in the extracellular matrix protein eyes shut homolog (EYS) are a common cause of retinitis pigmentosa, a blinding disease characterized by photoreceptor degeneration. EYS binds to matriglycan, a carbohydrate modification on O-mannosyl glycan substitutions of the cell-surface glycoprotein α-dystroglycan. Patients with mutations in enzymes required for the biosynthesis of matriglycan exhibit syndromic retinal atrophy, along with brain malformations and congenital muscular dystrophy. Protein O-mannosyltransferase 2 (POMT2) is an enzyme required for the synthesis of O-mannosyl glycans. To evaluate the roles of O-mannosyl glycans in photoreceptor health, we generated *protein O-mannosyltransferase 2 (pomt2)* mutant zebrafish by CRISPR. *pomt2* mutation resulted in a loss of matriglycan and abolished binding of EYS protein to α-dystroglycan. Mutant zebrafish presented with hydrocephalus and hypoplasia of the cerebellum, as well as muscular dystrophy. EYS protein was enriched near photoreceptor connecting cilia in the wild-type, but its presence and proper localization was significantly reduced in mutant animals. The mutant retina exhibited mis-localization of opsins and increased apoptosis in both rod and cone photoreceptors. Immunofluorescence intensity of G protein subunit alpha transducin 2 (GNAT2) antibody (a general cone marker) and 1D4 antibody (a long double cone marker) in mutant retinas did not differ from wild-type retinas at 1-month post fertilization, but was reduced at 6 months post fertilization, indicating significant cone degeneration. These data suggest that POMT2-mediated O-mannosyl glycosylation is required for EYS protein localization to the connecting cilium region and photoreceptor survival.

## 1. Introduction

Retinitis pigmentosa (RP) is a progressive blinding disease that affects 1 in 3000 to 4000 people worldwide with no effective therapy. This neurodegenerative condition is caused by mutations in more than 70 genes and is characterized by photoreceptor degeneration that typically begins during childhood. Retinitis pigmentosa 25 (RP25, OMIM#612424) is a common autosomal recessive form of the disease caused by mutations in eyes shut homolog (EYS) that result in rod, and eventually cone, degeneration [1,2,3,4,5,6,7,8,9]. In rare cases, EYS mutations can also cause cone-rod dystrophy, in which cones degenerate before rods [10]. EYS is a secreted extracellular matrix protein composed of multiple epidermal growth factor (EGF) and laminin globular (LG) domains.

How EYS contributes to photoreceptor health is poorly understood. EYS mutations in Drosophila cause abnormal development of ommatidia but do not cause photoreceptor degeneration in the fly [11,12]. Because some mammalian clades, including rodents, have lost the EYS locus [1], mutant zebrafish lines have been generated to explore EYS function [13,14,15]. EYS deficiency in zebrafish causes photoreceptor degeneration [13,14,15]. In the retinas of zebrafish and primates, EYS protein is localized near the connecting cilium/transition zone (CC/TZ) of photoreceptors [13]. EYS deficiency in zebrafish causes structural disruptions of the ciliary pocket [13] suggesting that EYS may play a role in the function of connecting cilia.

Recently, we have shown that EYS localization to the connecting cilium region is controlled by binding to matriglycan, a unique carbohydrate modification on α-dystroglycan (DG) [16]. Matriglycan is a repeating disaccharide structure [–3-xylose–α1,3-glucuronic acid-β1–]_n_ [17,18] extended onto O-mannosyl glycan substitutions. α-dystroglycan (α-DG) is a cell surface glycoprotein that binds to transmembrane β-DG, which interacts with the cytoskeleton. α-DG binds to laminin globular (LG) domains of several extracellular matrix (ECM) proteins including laminins [19,20,21], agrin [22], perlecan [23], neurexin-1 [24], Slit2 [25], and pikachurin [26,27,28] via its carbohydrate modification, matriglycan. Mutations in the enzymes involved in the biosynthesis of matriglycan cause syndromic retinal atrophy, that also manifest as congenital muscular dystrophy and brain malformation (reviewed in [29]). Interestingly, hypomorphic mutations in one of the enzymes required for matriglycan synthesis called protein O-mannose N-acetylglucosaminyltransferase 1 (POMGnT1) are found in several cases of non-syndromic retinitis pigmentosa (RP76, OMIM#617123) [30,31]. EYS-matriglycan binding is essential for targeting EYS protein to the connecting ciliary region because *pomgnt1* mutation in zebrafish results in a diminished level of matriglycan and mis-localization of EYS protein, suggesting the importance of EYS-matriglycan interactions in photoreceptor health. O-mannosyl glycosylation is initiated by the protein O-mannosyltransferase 1 and 2 (POMT1/2) complex in the endoplasmic reticulum (ER) [32]. Patients with null mutations of POMT2 do not survive beyond childhood; thus, the role of POMT2 in photoreceptor survival has not yet been established [33]. In this study, we show that deletion of POMT2 in zebrafish results in a loss of matriglycan, diminished expression and mis-localization of EYS, and photoreceptor degeneration.

## 2. Results

### 2.1. Pomt2 Mutation in Zebrafish Caused Diminished Expression of Matriglycan and EYS Binding

**Figure 1:**
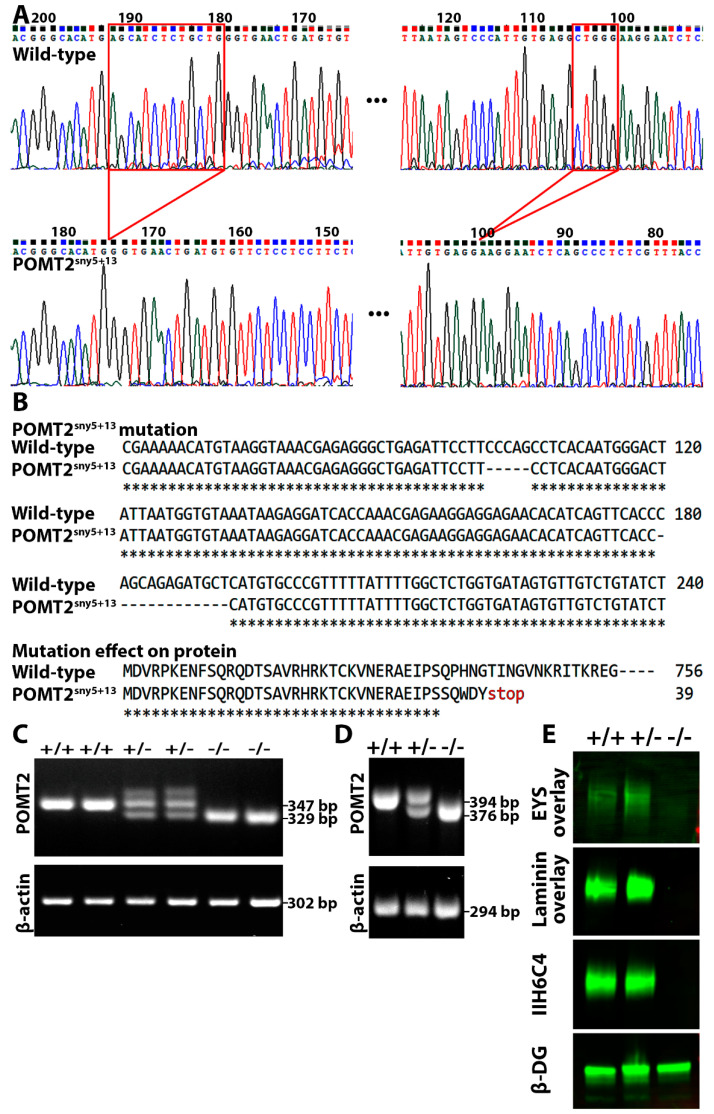
Deletion of *pomt2* in zebrafish ablated matriglycan and caused loss of EYS binding. *pomt2* mutant zebrafish were generated by CRISPR. Crossing of heterozygous mutant zebrafish generated homozygous mutant fish. Skeletal muscle lysates were used for IIH6C4 immunoblotting and EYS overlay assays. (**A**) Sequencing chromatogram of wild-type and *pomt2^sny5+13^* PCR product. The two small deletions in exon 2 are bracketed with red marks. (**B**) Alignment of part of exon 2 sequences showing the two small deletions in the *pomt2^sny5+13^* mutant and alignment of wild-type POMT2 protein with the expected truncated POMT2 peptides in the *pomt2^sny5+13^* mutant. The mutant POMT2 protein has only 39 amino acid residues in total. (**C**) Example of PCR genotyping showing that wild-type zebrafish produce a 347 bp fragment while homozygous mutant animals produce a 329 bp fragment. (**D**) RT-PCR of wild-type animals produce only a 394 bp fragment, homozygous mutant animals produce a 376 bp fragment, and heterozygous animals produce both 394 and 376 bp fragments. (**E**) IIH6C4 and β-DG Western blotting and EYS and laminin overlay on *pomt2^sny5+13^* mutant. Note abolished IIH6C4 reactivity and EYS and laminin binding in muscle lysate of homozygous *pomt2* mutant.

To study the roles of POMT2 in photoreceptor survival, *pomt2* mutant zebrafish, *pomt2^sny5+13^*, were generated by clustered regularly interspaced short palindromic repeats (CRISPR) genome editing (Figure 1A). The *pomt2^sny5+13^* allele had two small deletions in exon 2: nucleotides 101–105 (5 bp) and nucleotides 180–192 (13 bp) from the initiation codon. The 5-bp deletion generated a frameshift mutation that resulted in a premature stop codon before the second deletion. This mutation theoretically produced a severely truncated peptide, Q35S4X. Only 34 of the N-terminal amino acid residues were identical to the 756 amino acid residue wild-type POMT2 protein (Figure 1B). A pair of primers was designed to genotype the animals. Wild-type and mutant PCR products were 347 and 329 bps long, respectively (Figure 1C). To confirm that this mutation is null, we first carried out RT-PCR. While the wild-type produced a 394 bp product, the homozygous *pomt2* mutant animals produced the expected 376 bp truncated product (Figure 1D). Because an antibody that recognizes zebrafish POMT2 protein is not available, we evaluated the loss of O-mannosyl glycosylation in homozygous animals by Western blotting with IIH6C4 antibody, a monoclonal antibody that recognizes matriglycan [17,19], using skeletal muscle lysates extracted from *pomt2* mutant zebrafish. Wild-type and heterozygous animals were used as controls. As expected, wild-type and heterozygous zebrafish showed strong IIH6C4 immunoreactivity. However, IIH6C4 immunoreactivity was not detected in lysates from *pomt2* mutant zebrafish (Figure 1E). To evaluate the impact on ligand binding, overlay assays with EYS-5LG, a recombinant human EYS fragment containing all 5 LG domains capable of binding to matriglycan [16], and laminin-111 were performed. Binding for both EYS-5LG and laminin-111 was detected for wild-type and heterozygous lysates. However, EYS-5LG and laminin-111 binding was not detected in lysates from *pomt2* mutant zebrafish (Figure 1E). The β-DG control showed equal immunoreactivity across wild-type, heterozygous and mutant zebrafish lysates (Figure 1E). These results indicate that *pomt2* mutant zebrafish exhibit diminished expression of matriglycan on α-DG and loss of EYS and laminin binding activity.

### 2.2. Pomt2 Mutant Zebrafish Exhibited Hydrocephalus, Hypoplasia of the Cerebellum, and Muscular Dystrophy

The crossing of *pomt2* heterozygous animals yielded *pomt2* homozygous mutant embryos at the expected Mendelian ratio of 25%, when genotyped from 7 days post fertilization (dpf) to 21-dpf (Table 1). One month post fertilization (mpf), *pomt2* mutant zebrafish began dying at an increased rate relative to that of heterozygous and wild-type zebrafish, evident by lower-than-expected numbers of *pomt2* homozygous animals. Indeed, the mutant animals encompassed more than 25% of the 31 animals that spontaneously died between 3- and 5-weeks post fertilization (Table 2) (*p* = 0.042, Chi-square analysis). These data suggest increased lethality in the *pomt2* mutant. We also measured the body length and weight of all fish from several clutches at 1-mpf. There was no significant difference in the body length and weight between wild-type and heterozygous animals. Thus, wild-type and heterozygous animals were combined into one group for analyses. The average body length of wild-type/heterozygous animals was 8.52 ± 0.21 mm (Mean ± SEM). However, the body lengths of homozygous mutant animals were significantly shorter, with an average of 6.84 ± 0.35 mm (Mean ± SEM, *p* = 0.00023, Student’s *t*-test). Similarly, the mean body weight of wild-type/heterozygous animals was 8.80 ± 1.14 mg (Mean ± SEM), while that of the mutant animals was markedly smaller, at an average of 3.43 ± 0.73 mg (Mean ± SEM, *p* = 0.00015, Student’s *t*-test). These data indicate that while *pomt2* mutant zebrafish were viable, the mutation severely increased lethality. To avoid possible complications from malnutrition in phenotypic analysis, homozygous mutant zebrafish with feeding behavior similar to the wild-type animals and that actively swam without any sign of distress were selected for histological analysis of the retina, brain, and skeletal muscle. In all experiments described hereafter, the homozygous mutant zebrafish and wild-type or heterozygous controls were of the same age and of comparable body size.

**Figure 2:**
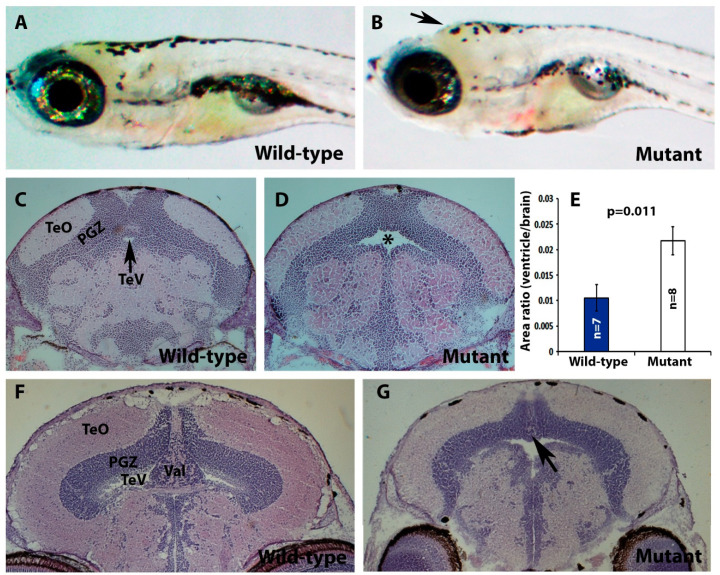

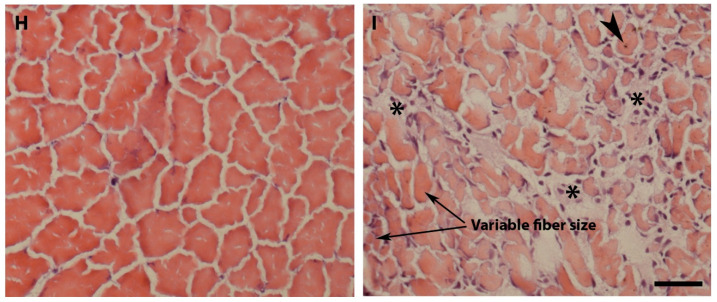
*pomt2* mutant zebrafish exhibit hydrocephalus, hypoplasia of the cerebellum, and muscular dystrophy. Sections of the brain and skeletal muscle were stained with H&E. (**A**,**B**) Lateral view of a wild-type and a *pomt2* mutant zebrafish both dead 3 weeks post fertilization. Note the domed head in the mutant (arrow). (**C**,**D**) H&E staining of the rostral region of the optic tectum in zebrafish at 2-mpf. Note *pomt2* mutant zebrafish showing an enlarged tectal ventricle (asterisk in (**D**)). (**E**) Ratio of ventricular area to total brain cross-sectional area. Note the increased ratio in the mutant. (**F**,**G**) H&E staining of the caudal region of the optic tectum in zebrafish at 2-mpf. Homozygous *pomt2^sny5+13^* mutant exhibited hypoplasia of the cerebellum (arrow). (**H**,**I**) H&E of skeletal muscle at 2-mpf. Note presence of dystrophic myofibers including necrotic fibers (asterisks), centrally located nuclei (arrowhead), and variably sized fibers. Scale bar in I: 50 µm for (**C**,**D**), 60 µm for (**F**,**G**), 28 µm for (**H**,**I**). Abbreviations: TeO = tectum opticum; PGZ = periventricular gray zone; TeV = tectal ventricle; Val = valvular cerebellum.

In addition to retinal atrophy, *POMT2* mutation in human patients causes type II lissencephaly, hydrocephalus, and hypoplasia of the cerebellum, as well as muscular dystrophy [33]. The heads of some of *pomt2* mutant zebrafish appeared domed in shape, consistent with a hydrocephalus phenotype (arrow in Figure 2B and compare with Figure 2A). Indeed, H&E staining of coronal sections of the brain revealed that the tectal ventricle (TeV) of *pomt2* mutant zebrafish was enlarged (asterisk, Figure 2D) when compared to the wild-type clutch mate (Figure 2C). To quantify this effect, we measured the area of zebrafish tectal ventricles and brain matter using ImageJ. We then calculated the ratio of the cross-sectional area of the tectal ventricle to the total brain cross-sectional area and found that this ratio was significantly greater in *pomt2* mutant zebrafish (Figure 2E). Apparent hypoplasia of the valvular cerebellum in *pomt2* mutant animals was observed as well (*n* = 8, Figure 2G, arrow) when compared to wild-type animals (Figure 2F). H&E staining of skeletal muscle also revealed that *pomt2* mutant zebrafish exhibited characteristics of muscular dystrophy (Figure 2I) including dystrophic fibers (asterisks), centrally located nuclei (arrowhead), and variations in fiber size (arrows). These data indicate that *pomt2* mutant zebrafish exhibit histological abnormalities of the brain in addition to muscular dystrophy.

### 2.3. EYS Protein Was Reduced and Mislocalized in the Pomt2 Mutant Retina

**Figure 3:**
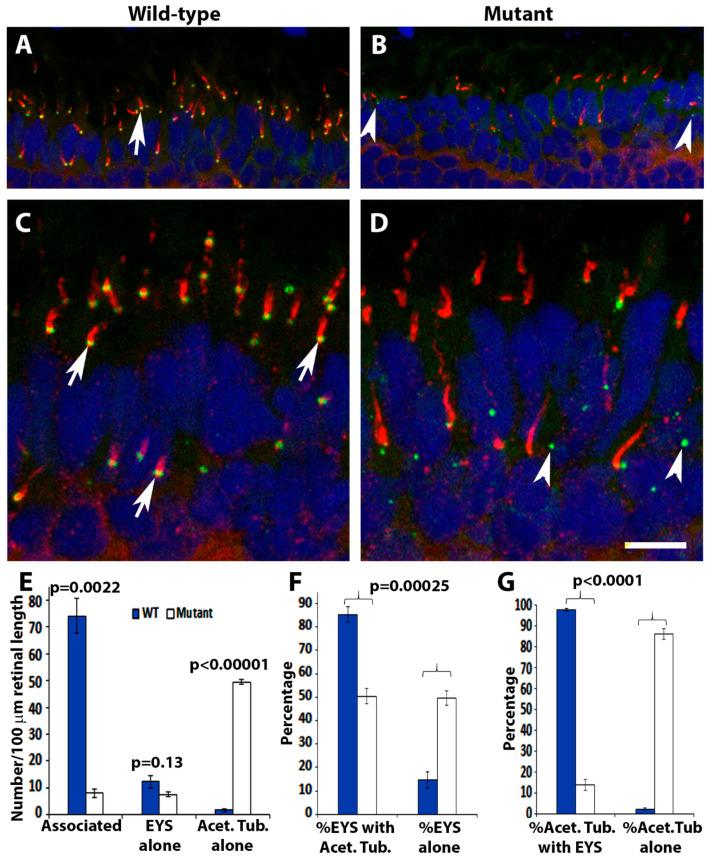
EYS protein was reduced and mis-localized in *pomt2* mutant zebrafish retinas. Retinal sections from zebrafish at 1-mpf were double immunostained with EYS (green fluorescence) and acetylated α-tubulin (red fluorescence). The sections were counterstained with DAPI to show nuclei. (**A**,**C**) Wild-type retina at 1-mpf. Most EYS-positive puncta in the wild-type retina were associated with the basal end of acetylated α-tubulin reactivity (arrows). (**B**,**D**) *pomt2^sny5+13^* homozygous mutant retina at 1-mpf. Overall, EYS immunoreactivity in the mutant was much lower than the wild-type. Most of the EYS-positive puncta were not associated with acetylated α-tubulin but were localized to the outer nuclear layer. (**E**) Counting of EYS-acetylated α-tubulin double staining from the outer nuclear layer to the retinal pigment epithelium (RPE) (*n* = 3). Note that acetylated α-tubulin reactivity associated with EYS puncta were decreased in the mutant, and acetylated α-tubulin not associated with EYS puncta were increased in the mutant. (**F**) Percentage of EYS associated with α-tubulin immunoreactivity and not associated with acetylated α-tubulin (*n* = 3). Note that EYS immunoreactivity associated with acetylated α-tubulin was reduced in *pomt2* mutant retinas, but non-associated EYS puncta were increased in these animals. (**G**) Percentage of acetylated α-tubulin immunoreactivity associated and not associated with EYS (*n* = 3). Note that acetylated α-tubulin immunoreactivity associated with EYS was reduced but acetylated α-tubulin immunoreactivity not associated with EYS was increased in *pomt2* mutant retinas. Scale bar in (**D**): 10.5 µm for (**A**,**B**); 5 µm for (**C**,**D**).

Deletion of POMGnT1 in zebrafish resulted in mis-localization of EYS protein in the retina [16]. Similarly, we evaluated EYS protein expression in *pomt2* mutant retinas by immunofluorescence staining. In 1-mpf wild-type retinas, anti-EYS puncta were observed at the connecting cilium region of photoreceptors located at the basal end of acetylated α-tubulin immunoreactivity, as expected [13] (arrows, Figure 3A,C). However, in homozygous *pomt2* mutant retinas, EYS immunoreactivity was significantly reduced by 1-mpf (Figure 3B,D). The number of EYS immunoreactive puncta per 100 µm retinal length was 15.68 ± 2.46 (Mean ± SEM) in the mutant retina, while the number for the wild-type was 86.45 ±4.52 (Mean ± SEM) (*p* < 0.0001, Student’s *t*-test). In the wild-type retina, most anti-EYS puncta were located at the basal end of acetylated α-tubulin immunoreactivity. In the mutant retina, however, most of these puncta appeared to be within the outer nuclear layer (arrowheads in Figure 3B,D). To quantify this effect, acetylated α-tubulin-immunoreactive axonemes associated with EYS puncta (Associated), acetylated α-tubulin-immunoreactive axonemes without EYS puncta associated (Acetylated tubulin alone), and EYS puncta not associated with acetylated α-tubulin (EYS alone) were counted on confocal images captured from the dorsal and ventral retina at 1-mpf. The number of axonemes associated with EYS puncta, in homozygous *pomt2* mutant retinas, were significantly reduced in numbers while the number of axonemes not associated with EYS puncta were significantly increased (Figure 3E). In *pomt2* mutant retinas, the proportion of EYS not associated with acetylated α-tubulin was increased while the proportion of EYS associated with acetylated α-tubulin was decreased (Figure 3F). Similarly, the proportion of acetylated α-tubulin not associated with EYS was increased while the proportion of acetylated α-tubulin associated with EYS was decreased (Figure 3G). These results indicate that EYS protein is reduced and largely mis-localized in *pomt2* mutant retinas.

### 2.4. Pomt2 Mutant Zebrafish Exhibited Photoreceptor Degeneration

**Figure 4:**
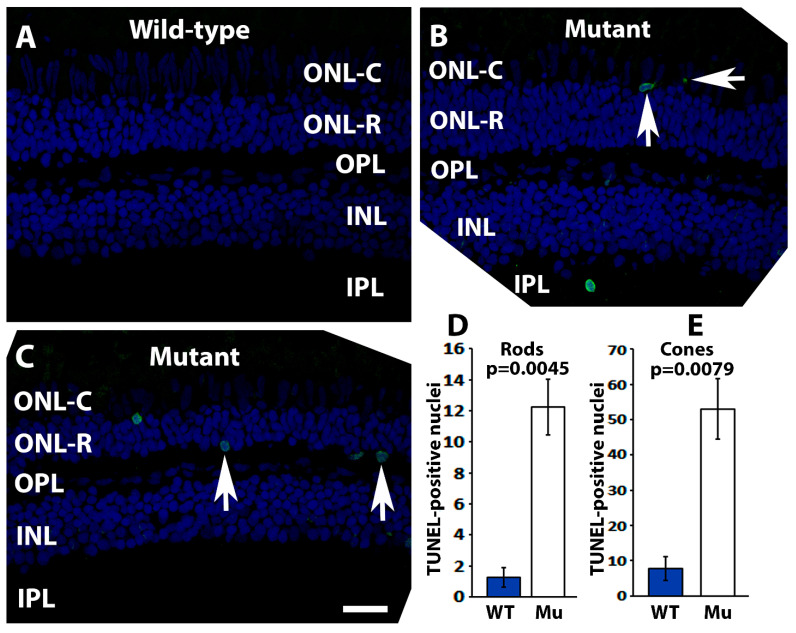
The quantity of TUNEL-positive photoreceptor nuclei was increased in *pomt2* mutant retinas. A TUNEL assay was carried out on zebrafish retinal sections at 6-mpf (green fluorescence). The sections were counter-stained with DAPI (blue fluorescence). (**A**) Wild-type retina. (**B**) *pomt2* mutant retina. Note examples of TUNEL-positive cone nuclei. (**C**) *pomt2* mutant retina. Note examples of TUNEL-positive rod nuclei. (**D**) Quantification of TUNEL-positive rod nuclei (*n* = 4). The number of TUNEL-positive rod nuclei was significantly increased in *pomt2* mutant retinas compared to the controls. (**E**) Quantification of TUNEL-positive cone nuclei (*n* = 4). The number of TUNEL-positive cone nuclei was significantly increased in *pomt2* mutant retinas compared to the controls. Scale bar in C: 30 µm.

*pomgnt1* mutation in zebrafish causes photoreceptor degeneration [16]. Likewise, EYS mutant zebrafish also manifest photoreceptor degeneration [13,14,15]. Loss of EYS in the connecting cilium region in *pomt2* mutant zebrafish may ultimately cause photoreceptor degeneration. Thus, a terminal deoxynucleotidyl transferase dUTP nick end labeling (TUNEL) fluorescence assay was carried out to confirm the presence of apoptotic cells in the *pomt2* mutant retina (Figure 4). In the wild-type retina, TUNEL-positive nuclei were rarely observed (Figure 4A). In *pomt2* mutant retinas, however, the number of TUNEL-positive nuclei were increased in the cone cell layer (ONL-C) (Figure 4B, arrows) as well as in the rod cell layer (ONL-R) (Figure 4C, arrows). Quantitative analysis showed that the numbers of TUNEL-positive cone and rod nuclei were significantly increased in *pomt2* mutant retinas when compared to the wild-type (Figure 4D, Student’s *t*-test). This indicated increased cell death in both cone and rod photoreceptors in *pomt2* mutant retinas.

**Figure 5:**
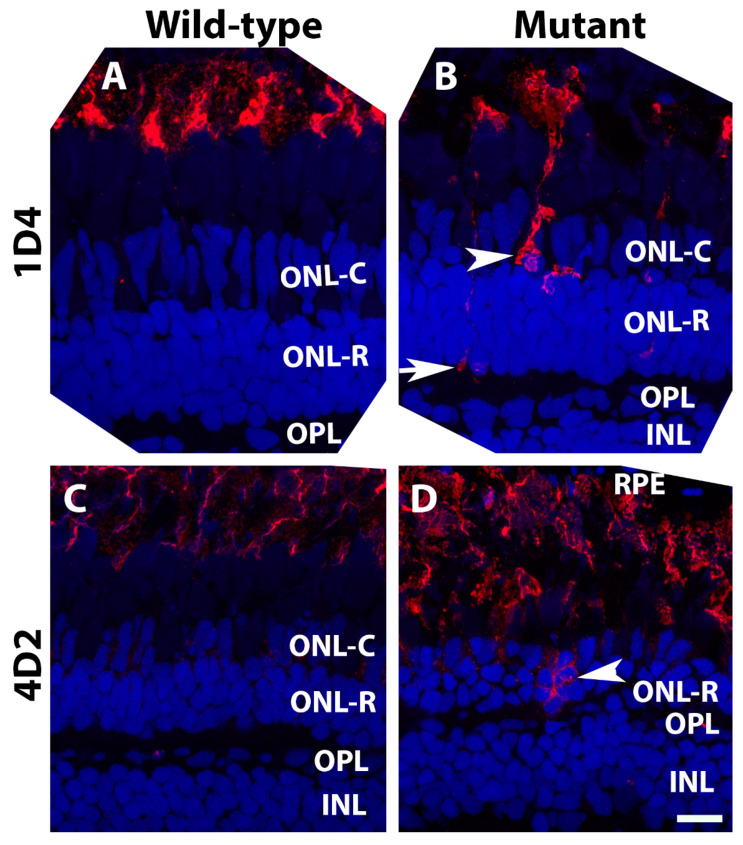
Opsin mis-localization was found in *pomt2* mutant retinas. Retinal sections from zebrafish at 6-mpf were immunostained with antibodies 1D4 and 4D2 (red fluorescence) and counter-stained with DAPI (blue) (*n* = 3). (**A**) 1D4 immunostaining of a wild-type retina at 6-mpf. (**B**) 1D4 immunostaining of a homozygous *pomt2^sny5+13^* mutant retina at 6-mpf. Note the presence of strongly 1D4-stained cell bodies (arrowhead) and terminals (arrow). (**C**) 4D2 immunostaining of a wild-type retina at 6-mpf. (**D**) 4D2 immunostaining of a homozygous *pomt2^sny5+13^* mutant retina at 6-mpf. Note strongly 4D2 fluorescent cell bodies in the ONL. Scale bar in (**D**): 10 µm.

A hallmark of photoreceptor degeneration is the mis-localization of outer segment proteins to other cellular compartments of the photoreceptor. Antibody 1D4, which labels red opsin in zebrafish [34], strongly stained the outer segment layer of wild-type retinas with minimal staining of the photoreceptor cell bodies (Figure 5A). In *pomt2* mutant retinas, however, strongly labeled cell bodies (Figure 5B, arrowhead) and terminals (Figure 5B, arrow) were frequently observed. Similarly, rhodopsin antibody, 4D2, intensely labeled rod outer segments but weakly labeled the outer nuclear layer of wild-type retinas (Figure 5C). In *pomt2* mutant animals, however, strong 4D2 reactivity was frequently found in the outer nuclear layer (Figure 5D, arrowhead). Together, these results suggest mis-localization of opsins in *pomt2* mutant photoreceptors.

**Figure 6:**
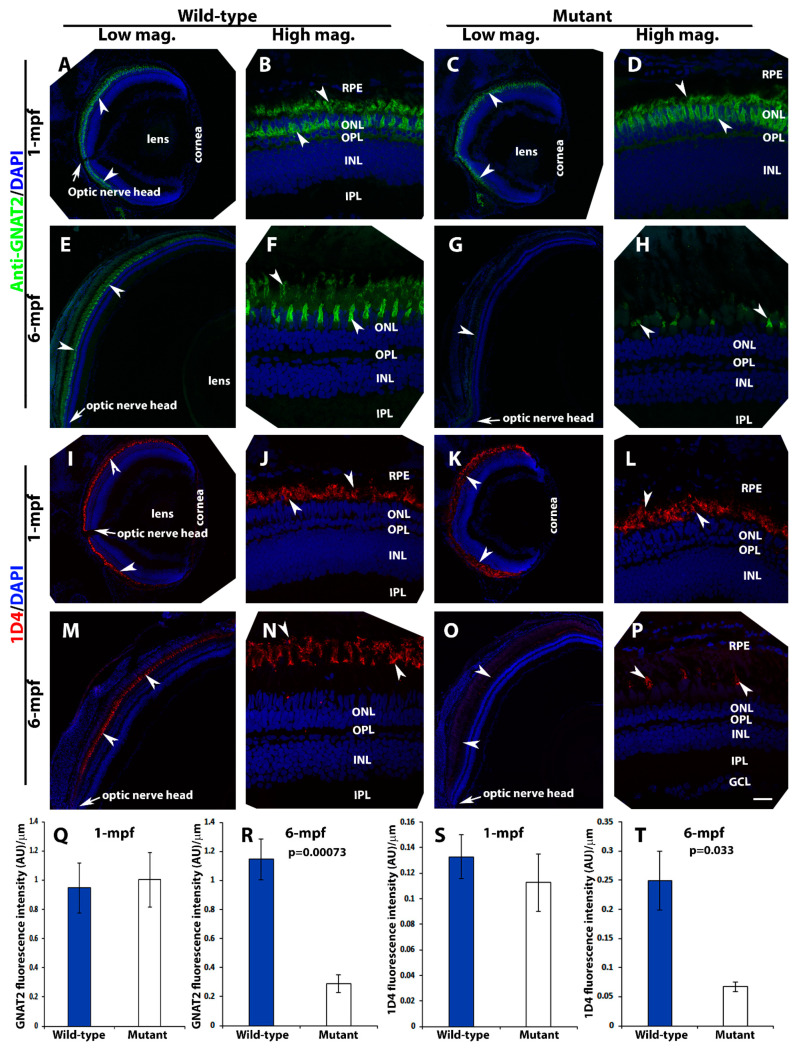
Loss of photoreceptors in *pomt2* mutant retinas at 6-mpf but not 1-mpf. Zebrafish retinal sections at 1-mpf and 6-mpf were immunostained with GNAT2 antibody (green fluorescence, **A**–**H**) and 1D4 (red fluorescence, **I**–**P**). The sections were counter-stained with DAPI. To quantify loss of cone photoreceptors, GNAT2 and 1D4 immunoreactive intensities were measured (**Q**–**T**). (**A**,**B**) Wild-type GNAT2 immunostaining at 1-mpf. (**C**,**D**) Homozygous *pomt2^sny5+13^* mutant GNAT2 immunostaining at 1-mpf showing similar immunoreactivity compared to the wild-type. (**E**,**F**) Wild-type GNAT2 immunostaining at 6-mpf. (**G**,**H**) Homozygous *pomt2^sny5+13^* mutant GNAT2 immunostaining at 6-mpf showing reduced immunoreactivity compared to the wild-type. (**I**,**J**) Wild-type 1D4 immunostaining at 1-mpf. (**K**,**L**) Homozygous *pomt2^sny5+13^* mutant 1D4 immunostaining at 1-mpf showing similar immunoreactivity compared to the wild-type. (**M**,**N**) Wild-type 1D4 immunostaining at 6-mpf. (**O**,**P**) Homozygous *pomt2^sny5+13^* mutant 1D4 immunostaining at 6-mpf showing reduced immunoreactivity compared to the wild-type. (**Q**) GNAT2 immunofluorescence intensity (artificial units, AU) quantification of wild-type and mutant fish at 1-mpf (*n* = 3). There was no significant difference between wild-type and mutant retinas. (**R**) GNAT2 immunofluorescence intensity quantification of wild-type and mutant fish at 6-mpf (*n* = 3). GNAT2 immunoreactivity was significantly reduced in mutant retinas. (**S**) 1D4 immunofluorescence intensity quantification of wild-type and mutant fish at 1-mpf (*n* = 3). There was no significant difference between wild-type and mutant retinas. (**T**) 1D4 immunofluorescence intensity in the mutant retina was reduced at 6-mpf (*n* = 3). Arrowheads indicate examples of GNAT2 immunoreactivity (A-H) and 1D4 immunoreactiity (I-P). Arrows indicate location of optic nerve heads. Scale bar in P: 100 µm for all low magnification panels and 18.7 µm for all high magnification panels.

To evaluate photoreceptor degeneration further, immunostaining with anti-G protein subunit alpha transducin 2 (GNAT2) (a general cone marker) and anti-1D4 (a long-double cone marker) was carried out on retinal sections at 1-mpf and 6-mpf. The immunoreactivity pattern in anti-GNAT2 stained homozygous *pomt2* mutant retinas at 1-mpf (Figure 6C,D) was very similar to patterns observed in the wild-type retina of the same age (Figure 6A,B). Likewise, immunoreactivity patterns from 1D4 staining in homozygous *pomt2* mutant retinas at 1-mpf (Figure 6K,L) were very similar in the wild-type retina (Figure 6I,J)). At 6-mpf, however, GNAT2 immunoreactivity in homozygous *pomt2* mutant zebrafish was reduced (compare Figure 6G,H to Figure 6E,F). Similarly, 1D4 immunoreactivity was significantly reduced in homozygous *pomt2* mutant zebrafish when compared to the wild-type zebrafish at 6-mpf (compare Figure 6O,P to Figure 6M,N). Semi-quantitative measurements of anti-GNAT2 and 1D4 reactivity showed that the immunofluorescence intensities of these antibodies were similar between wild-type and *pomt2* mutant retinas at 1-mpf (Figure 6Q,S). However, the immunofluorescence intensity for both antibodies was reduced in the *pomt2* mutant retina at 6-mpf (Figure 6R,T). To determine whether reduction of anti-GNAT2 and 1D4 reactivity in *pomt2* mutant animals was a result of loss of cones, we counted the number of DAPI-labeled cone nuclei from confocal images of dorsal retina near the optic nerve head at 1- and 6-mpf. At 1-mpf, there was no difference in the number of cone nuclei between the wild-type (38.13 ± 1.54 cones/100 µm retina, mean ± SEM) and the *pomt2* mutant animals (34.32 ± 0.09 cones/100 µm retina, *p* = 0.264, Student’s *t*-test). However, at 6-mpf, there was a significant decrease in the number of cone nuclei in *pomt2* mutant (15.12 ± 0.79 cones/100 µm retina) from the wild-type zebrafish (31.91 ± 1.51 cones/100 µm retina, *p* = 0.0022, Student’s *t*-test). These data indicated that there was a loss of cone photoreceptors in *pomt2* mutant zebrafish at 6-mpf. We also counted rod nuclei from the same images. The number of rod nuclei between wild-type (27.89 ± 0.59 rods/100 µm retina) and *pomt2* mutant zebrafish (28.02 ± 0.65 rods/100 µm retinas, *p* = 0.94, Student’s *t*-test) were similar at 1-mpf, as expected. Unlike cone photoreceptors, however, there was no difference in the number of rod nuclei at 6-mpf between the wild-type (57.41 ± 2.58 rods/100 µm retina) and *pomt2* mutant (57.48 ± 1.98 rods/100 µm retina) animals (*p* = 0.99, Student’s *t*-test). Together, the reduction in anti-GNAT2 and 1D4 reactivity and in the number of cone nuclei in *pomt2* mutant animals at 6-mpf indicate that cone photoreceptors degenerate. While mis-localization of 4D2 and an increase in the number of TUNEL-positive rod nuclei in *pomt2* mutant retina indicate rod degeneration, lack of significant difference in the number of rod nuclei from the wild-type suggests that the rods degenerate at a much slower pace.

## 3. Discussion

Localization of EYS to the photoreceptor connecting cilium is critical for photoreceptor survival. In this manuscript, we showed that deletion of POMT2 in zebrafish resulted in the ablation of matriglycan and abolished EYS binding to α-DG. Diminished matriglycan in *pomt2* mutant zebrafish resulted in a reduced EYS protein level in the retina and a failure of EYS localization to the connecting cilium region. While photoreceptor development was not affected in *pomt2* mutant zebrafish, photoreceptor density was significantly reduced by 6-mpf. Apoptosis of both cone and rod photoreceptors in the *pomt2* mutant retina was significantly increased. These results indicate that POMT2 is essential for EYS protein localization near connecting cilia and for photoreceptor survival.

EYS protein is highly enriched near the connecting cilium in zebrafish [13,15]. We have previously shown that EYS binds to matriglycan and that this binding is required for normal EYS protein localization in the retina [16], as POMGnT1 mutation in zebrafish diminished the level of matriglycan and EYS protein at the connecting ciliary region. Most of the mis-localized EYS protein in the *pomgnt1* mutant retina was located within secretory vesicles, as ectopic EYS immunoreactive puncta were co-localized with synaptotagmin-1-positive puncta (a secretory vesicle/v-SNARE marker). Mutations in POMT2 often cause Walker-Warburg syndrome, whereby afflicted patients die during early childhood. Thus, it has not yet been established whether POMT2 mutations cause degeneration of photoreceptors. In our study, *pomt2* mutant zebrafish exhibited loss of reactivity to IIH6C4 antibody, diminished EYS binding to α-dystroglycan, and reduced EYS protein at photoreceptor connecting cilia. In addition, EYS immunofluorescence labeling also showed a significant reduction in EYS protein level in the *pomt2* mutant zebrafish that appeared more pronounced than in *pomgnt1* mutant zebrafish [16]. We speculate that in the absence of matriglycan, some EYS proteins that fail to be localized near connecting cilia may be degraded. Furthermore, photoreceptor density in *pomt2* mutant zebrafish was decreased at 6-mpf but not at 1-mpf. These data indicate that POMT2 is essential for photoreceptor survival, providing further support for the importance of the EYS-matriglycan interaction in photoreceptor health.

Synthesis of O-mannosyl glycans is initiated by the transfer of mannose to Ser/Thr residues on α-DG by the POMT1/2 complex. POMGnT1 catalyzes the addition of a β1,2-linked N-acetylglucosamine off the O-linked mannose (reviewed in [29]). POMGnT1 activity is required for efficient synthesis of matriglycan by like-glycosyltransferase (LARGE) [35] or LARGE2 [36] in the Golgi onto the O-mannosyl glycan substitutions. Mutations in the enzymes of this glycosylation pathway cause syndromic retinal atrophy, brain malformations, and muscular dystrophy. Namely, POMGnT1 null mutations often cause muscle-eye-brain disease, a syndromic retinal atrophy that also manifests as brain malformations and muscular dystrophy [7]. Interestingly, hypomorphic mutations in POMGnT1 have been found in retinitis pigmentosa (RP76) patients [30,31] without brain malformations and muscular dystrophy, supporting that O-mannosyl glycosylation deficiency causes photoreceptor degeneration in humans. Hypomorphic mutations in other enzymes involved in matriglycan biosynthesis may also be found in patients with non-syndromic photoreceptor degeneration in the future.

Similar to laminin α chains, EYS has five LG domains. Binding of laminins to matriglycan is mediated through their LG 4 and 5 domains [37]. Sequence alignment of LG domains between laminin α2 and EYS suggest that LG 4 and 5 of EYS had the greatest homology with LG domains 4 and 5 of laminin, suggesting that LG domains 4–5 of EYS may be involved in matriglycan binding. Further studies are required to determine the EYS domains required for binding to matriglycan and whether missense mutations in LG domains 4 and 5, D2767Y and D3028Y found in patients [38,39] affect matriglycan interaction.

Loss of matriglycan in *pomgnt1* and *pomt2* mutant zebrafish diminished EYS localization to photoreceptor connecting cilia. While EYS is not required for photoreceptor development and ciliogenesis [13], it is essential for photoreceptor survival in humans as well as in zebrafish. Interestingly, *eys* has been lost in certain mammalian clades such as the armadillo, little brown bat, guinea pig, rat and mouse [1]. It is not known whether an analogous protein compensates for the loss of EYS or photoreceptors adapted structurally and functionally to the absence of EYS in these species. Photoreceptor degeneration in *pomgnt1* and *pomt2* mutant zebrafish could be a direct effect from the loss of O-mannosyl glycans or an indirect effect from the secondary loss of EYS at the connecting cilium due to O-mannosyl glycan deficiency. Given that there is no evidence of photoreceptor degeneration in *Pomgnt1* mutant mice, our data are consistent with the idea that O-mannosyl glycosylation promotes photoreceptor survival by EYS-matriglycan binding, which facilitates EYS protein localization to the connecting cilium region. Future investigation should be aimed at elucidating how matriglycan helps traffic EYS protein to the connecting cilium region.

## 4. Materials and Methods

### 4.1. Zebrafish Maintenance

AB/Tubingen strain Zebrafish were grown in a water system at pH 6.6–7.4 around 26–28.5 °C fed once daily with Gemma Micro (Skretting, Tooele, UT, USA). The room was set with a daily light cycle of 14 h of light and 10 h of darkness. All experiments were performed in accordance with the National Institute of Health guidelines and approved by the Institutional Animal Care and Use Committee at the State University of New York Upstate Medical University.

### 4.2. Generation of the Pomt2^sny5+13^ Mutant Line

The zebrafish *has one* locus for *pomt2* on chromosome 17. Alignment of zebrafish and human POMT2 revealed that 67% of amino acid sequences were identical between the two orthologs. Clustered regularly interspaced short palindromic repeats (CRISPR)/Cas9 technology was used to generate the *pomt2* mutant. Two gRNAs targeting exon 2 of the zebrafish *pomt2 locus*, GTCCCATTGTGAGGCTGGGA (antisense strand), and GCCCGTTTTTATTTTGGCTC were synthesized at GenScript (Piscataway, NJ, USA). The gRNAs were co-injected with SpCas9 protein (New England Biolabs, Ipswich, MA, USA) to the zebrafish embryos at 1-cell stage. Genomic DNA extracted from tail-fin clips of surviving animals were used for PCR to amplify a 347 bp fragment spanning the gRNA target sites with primers forward TGAACTCTGCCATTGTGTTCA and reverse TGAACTCTGCCATTGTGTTCA. A founder animal that yielded amplicons that contained a smaller fragment was crossed with wild-type animals to obtain an F1 generation, which was screened by PCR with the same primers. The smaller sized was extracted from agarose gels and sequenced to identify the mutation and compared to the wild-type sequence. This effort yielded a mutant line containing two small deletions, one of 5 nucleotides and another of 13 nucleotides. We have named this mutant allele as *pomt2^sny5+13^* in accordance with the convention on zebrafish mutant line designation. This mutation occurred within exon 2 and is expected to disrupt the reading frame resulting in the loss of POMT2 protein (Figure 1).

### 4.3. RT-PCR

Skeletal muscle from 2-mpf zebrafish was used to extract total RNA using Rneasy Plus Mini Kit (QIAGEN, Germantown, MD, USA, Cat#74104). POMT2 RT-PCR was carried out with the iTaq Universal SYBR Green One-Step Kit (Bio-Rad, Hercules, CA, USA, Cat#1725150) with the following primers: forward ACCGTTACTGAACTCTGCCA and reverse TGTAATAGCTTCCCATTTTCCCA. Internal control β-actin RT-PCR primers were forward AGATCAAGATCATTGCTCCCC and reverse CCTTTGCCAGTTTCCGCATC.

### 4.4. Western Blotting Overlay Experiment

Total protein was extracted from the skeletal muscle of the zebrafish body wall with radio-immuno precipitation assay (RIPA) buffer (50 mM Tris, pH 8.0, 150 mM NaCl, 1.0% NP-40, 0.5% sodium deoxycholate, 0.1% SDS) containing a protease inhibitor cocktail. Twenty mg of total protein was resolved by SDS-PAGE and transferred to PVDF membranes. The membrane was blocked with Tris-buffered saline (50 mM Tris, pH 7.4, 150 mM NaCl) containing 1% BSA, followed by incubation with IIH6C4 (Santa Cruz, Dallas, TX, USA, Cat# sc-73586, 1:400 dilution), anti-β-dystroglycan (Abcam, Waltham, MA, USA, Cat# ab49515) in TBS with 0.2% Tween-20 and 1% BSA overnight at 4 °C with gentle shaking. The membrane was washed with TBST (TBS with 0.1% Tween 20) three times followed by incubation with the appropriate DyLight Fluor secondary antibodies wash three times in TBST and once with TBS and visualized with Odyssey CLx (Li-Cor Biosciences, Lincoln, NE, USA).

For EYS overlay, a pSecTag2A with N-terminal HA-tagged cDNA fragment encoding amino acid residues 1862–3144 of human EYS protein was transfected into HEK293 cells. This fragment consists of all 5 laminin G domains [16]. EYS conditioned medium was collected 2 days after transfection. Twenty mg of total proteins extracted from zebrafish skeletal muscle were separated on SDS-PAGE and transferred to PVDF membrane. The membrane was blocked with 3% BSA in Tris-buffered saline (50 mM Tris, pH 7.4, 150 mM NaCl, 1mM CaCl_2_, and 1 mM MgCl_2_) for 30 min. Then, the membrane was incubated with EYS-conditioned medium overnight at 4 °C. Detection of bound EYS with anti-HA was identical to the above procedure with the exception that all buffers contained 1 mM CaCl_2_ and 1 mm MgCl_2_.

For laminin overlay, laminin-111 (Millipore-Sigma, St. Louis, MO, USA) was used to incubate with the PVDF membrane and detected with laminin-111 antibody as above.

### 4.5. Immunofluorescence Staining

Whole heads were embedded in Tissue-Tek^®^ O.C.T. Compound (Sakura Finetek USA, Torrance, CA, USA), cryo-sectioned along the dorso-ventral plane, and mounted onto Fisherbrand Superfrost plus slides (Fisher Scientific, Hampton, NH, USA). After fixation with 4% paraformaldehyde for 10 min, the sections were permeabilized with 0.1% Triton X-100 in phosphate buffer and incubated with 3% bovine serum albumin (BSA) in 0.1M phosphate buffer (PB) at room temperature for 1 h. Primary antibodies against GNAT2 (MBL International, Woburn, MA, USA, Cat#PM075, 1:400), EYS (Novus Biological, Centennial, CO, USA, Cat# NBP1-90038, 1:300), acetylated α-tubulin (Millipore-Sigma, Cat#T6793, 1:1000), red opsin (1D4, Abcam, Cat#AB5417, 1:1000), and rhodopsin (4D2, Abcam, Cat#AB98887, 1:300) were applied to the sections and incubated in a humidified chamber overnight at 4 °C. The sections were washed with PB containing 0.1% Triton X-100 and incubated with appropriate FITC-conjugated anti-rabbit IgG (Jackson ImmunoResearch, West Grove, PA, USA, 1:300) or RITC-conjugated anti-mouse IgG (Jackson ImmunoResearch, 1:300) for 2 h at room temperature in a humidified chamber. The sections were then counter-stained with 4′,6-diamidino-2-phenylindole (DAPI) to label nuclei. After washing with PB three times, VECTASHIELD^®^ mounting medium (Vector Laboratories, Newark, CA, USA, Cat# H-1000) was applied to the sections. The sections were then covered with coverslips. Immunofluorescence was visualized with a Zeiss Axioskop epifluorescence microscope and a Leica SP8 confocal microscope. Epifluorescence images were captured with a mono 12-bit camera and Qcapture Pro 6.0 (Qimaging, Teledyne Photometrics, Tucson, AZ, USA).

To evaluate apoptosis in the retina, a terminal deoxynucleotidyl transferase dUTP nick end labeling (TUNEL) assay was performed using the ApopTag Fluorescein In Situ Apoptosis Detection Kit (Millipore-Sigma) according to the manufacturer’s suggestions. TUNEL-positive nuclei were imaged with a Leica SP8 confocal microscope.

### 4.6. Semi-Quantitative Analyses Immunofluorescence Staining Signals

To quantify EYS localization near the connecting ciliary region, EYS-positive puncta associated with the basal end of acetylated α-tubulin immunoreactivity, EYS-positive puncta not associated with the basal end of acetylated α-tubulin immunoreactivity, and acetylated α-tubulin immunoreactivity not associated with EYS puncta, were counted from confocal images captured from EYS and acetylated α-tubulin immunostained retinal sections. Data were analyzed by ANOVA. Post hoc comparisons were done by Student’s *t*-test with Bonferroni correction.

To quantify GNAT2 and 1D4 immunofluorescence reactivity, immunofluorescence was visualized with a Zeiss Axioskop epifluorescence microscope. Epifluorescence images were captured with a mono 12-bit camera and QCapture Pro 6.0 (QImaging) from the dorsal retina near the optic nerve head. Fluorescence intensity in the outer segment layer was measured with ImageJ. Fluorescence intensity in areas with no tissue was considered background and was subtracted. Data were analyzed by Student’s *t*-test.

## Figures and Tables

**Table 1:** Genotypes of animals from heterozygous crossings at 7-dpf to 6-mpf.

Age of Animals	+/+	+/−	−/−	*p* Value *
7-dpf	23	20	12	0.59
14-dpf	14	26	15	0.70
21-dpf	20	21	15	0.76
1-mpf	45	83	30	0.081
2-mpf	28	62	14	0.0066
3–6-mpf	169	264	38	2.13 × 10^−17^

* Chi-square analysis for homozygous animals expected at 25%.

**Table 2:** Genotypes of dead animals collected from heterozygous crossings between 21- to 35-dpf.

Age Range	+/+	+/−	−/−	*p* Value *
21 to 35 dpf	2	17	12	0.034

* Chi-square analysis for wild-type, heterozygous and homozygous animals expected at 25%, 50% and 25%, respectively.
